# Dielectrophoretic Manipulation and Separation of Microparticles Using Microarray Dot Electrodes

**DOI:** 10.3390/s140406356

**Published:** 2014-04-03

**Authors:** Bashar Yafouz, Nahrizul Adib Kadri, Fatimah Ibrahim

**Affiliations:** 1 Department of Biomedical Engineering, Faculty of Engineering, University of Malaya, 50603 Kuala Lumpur, Malaysia; E-Mails: bashar.yafouz@siswa.um.edu.my (B.Y.); fatimah@um.edu.my (F.I.); 2 Centre for Innovation in Medical Engineering (CIME), Faculty of Engineering, University of Malaya, 50603 Kuala Lumpur, Malaysia

**Keywords:** dielectrophoresis (DEP), microparticle separation, dot electrode, microfluidics, BioMEMS

## Abstract

This paper introduces a dielectrophoretic system for the manipulation and separation of microparticles. The system is composed of five layers and utilizes microarray dot electrodes. We validated our system by conducting size-dependent manipulation and separation experiments on 1, 5 and 15 μm polystyrene particles. Our findings confirm the capability of the proposed device to rapidly and efficiently manipulate and separate microparticles of various dimensions, utilizing positive and negative dielectrophoresis (DEP) effects. Larger size particles were repelled and concentrated in the center of the dot by negative DEP, while the smaller sizes were attracted and collected by the edge of the dot by positive DEP.

## 1. Introduction

Particle manipulation and separation techniques have been of interest to many research groups worldwide for various biomedical applications, including cell concentration, separation, patterning, trapping and positioning [1]. These methods have facilitated the development of cost-effective point-of-care (POC) devices that can rapidly collect, prepare and analyze human biological samples.

Diverse diagnostic approaches have been exploited to manipulate particles using lab-on-a-chip (LOC) platforms; however, dielectrophoresis (DEP), as the sample preparation stage of a POC device, presents unique features; including the differentiation between particles is based merely on the particles' dielectric properties (electrical conductivity and permittivity) determined by the phenotype of the respective particles, and the high selectivity and efficacy of particles manipulation on micro and nano levels [2].

DEP is a non-invasive method that describes the movement of polarizable particles when subjected to a non-uniform electric field. The basis for generating the DEP force is the interaction between the particle's dipole and the spatial gradient of the electric field [3]. DEP has been extensively employed as a potential technique to manipulate viruses [4], proteins [5], bacteria [6], DNA [7], spores [8], algae [9], parasites [10] and nano-sized latex particles [11]. In addition, DEP has been used in research on cell lysis [12] and to characterize a wide range of yeast cells [13] and mammalian cells, such as neurons [14], leukemia cells [15], platelets [16], cancer cells [17], and sperm cells [18].

An electrode generates the non-uniform electric field that develops the DEP effects. Previous electrode structures for early DEP studies were constructed from thin metal wires, needles or plates [19,20]. However, current DEP platforms utilize advanced microfabrication technologies to produce microelectrode arrays that are capable of generating strong DEP forces with small applied voltages [6,7,21].

Electrode structures can be generally classified into two categories: planar and three-dimensional (3D) [22]. Planar electrodes are commonly fabricated on the bottom of a microchannel utilizing lithography procedures, such as interdigitated [23], castellated [24], spiral [25], curved [26], oblique [27], quadrupole [28] and matrix [29]. In contrast, 3D electrodes are patterned on the bottom, both the bottom and the top or the sidewalls of the microchannel; however, these designs involve complex fabrication processes. Examples of 3D electrodes include a grid pattern [30], microwells [31], DEP wells [32], extruded patterns [33], a sidewall pattern [34] and a top-bottom pattern [35].

A wide range of different electrode designs has evolved for various research applications. For instance, interdigitated electrodes were designed to separate certain populations of particles based on their characteristic electrical properties, and grid electrodes were proposed to precisely control the physical movements of single cells [36]. In addition, 3D electrodes were developed to conduct characterization studies on large populations of particles. Hence, the objective of the investigation at hand dictates the electrode structure to be used.

In this paper, we introduce a dielectrophoretic system that utilizes microarray dot electrodes for the manipulation and separation of microparticles. The 4 × 4 microarray dot electrode geometry used in the current study followed the Fatoyinbo *et al.*, model, which used a similar electrode geometry to conduct cell characterization experiments on homogenous populations [37]. We propose adapting this electrode geometry to conduct separation experiments on a mixture of non-homogenous populations using polystyrene particles. In order to avoid the overlapping between the electric fields produced by neighbor dots, a ground plane was introduced in the electrode area between the dot apertures [38].

The dot electrode geometry possesses several advantages because it has a well-defined and confined region of analysis, effective electric field penetration and an axisymmetrical electric field distribution as shown in electric field simulations in [38]. As another benefit of this radial electrode geometry, neither field mapping nor image segmentation is required to measure the DEP force on the microparticles. The DEP force quantification depends on the change shifts in light transmission through the dot before and after electric potential application. In addition, the simple and rapid fabrication processes of planar electrodes make microarray dot electrode a potential solution to be utilized in research involving the development of cost-effective POC devices.

Moreover, we have implemented a novel LOC design to be compatible with the microarray dot electrode configuration. Most of the microarray electrodes often utilize horizontal DEP effect which is typically produced by a parallel arrangement of planar electrodes on the same layer. However, the nature of the LOC design in the current study, gold dot microarray electrode on the bottom layer while Indium Tin Oxide (ITO) counter electrode at the top, implies the generation of a vertical DEP effect in which the number of particles experiencing DEP force greatly increases.

As polystyrene microparticles have been widely used to evaluate the performance of DEP systems [26,39–43], we employed 1, 5 and 15 μm polystyrene particles to validate the proposed DEP method by conducting size-dependent manipulation and separation experiments on a mixtures of different-size microparticles.

## 2. Theory

DEP is a phenomenon that describes the force acting on dielectric particles suspended in non-uniform electric field. The magnitude of the particle movement and the direction of this movement depend on the relative polarizabilities of the particles and the surrounding medium [22]. For a spherical particle of radius *r*, the DEP force is defined as:

(1) 〈F→DEP〉=2πr3εoεmRe[K(ω)]∇E2

where ε*_o_* and ε*_m_* represent the permittivity values of the free space and the relative permittivity of the surrounding medium, respectively; ∇*E* denotes the electric field gradient; and Re[*K*(ω)] is the real part of the Clausius-Mossotti factor. The Clausius-Mossotti factor is given by:

(2) $$K(\omega )=\frac{\varepsilon_{p}^{*}-\varepsilon_{m}^{*}}{\varepsilon_{p}^{*}+2\varepsilon_{m}^{*}}$$

$\varepsilon_{P}^{*}$ and $\varepsilon_{m}^{*}$ in Equation (2) are the complex permittivities of particles and the suspending medium, respectively, where $\varepsilon *=\varepsilon –j\frac{\sigma }{\omega }$, ε the permittivity, σ the conductivity, $j=\sqrt{-1}$ and ω the angular frequency of the applied field.

Particles exhibit a positive or negative DEP (p-DEP or n-DEP) effect depending on the polarity of the Re[*K*(ω)] which value ranges between −0.5 and 1 for spherical particles. The Re[*K*(ω)] is a function of the applied frequency and the relative magnitudes of $\varepsilon_{P}^{*}$ and $\varepsilon_{m}^{*}$ [44]. By controlling these factors, target particles migration can be directed according to their DEP properties, enabling particles manipulation and separation. For example, when $\varepsilon_{P}^{*}$ is higher than $\varepsilon_{m}^{*}$, the Re[K(ω)] sign is positive, and the particles experience p-DEP and travel towards the higher electric field gradient region. On the contrary, when $\varepsilon_{P}^{*}$ is lower than $\varepsilon_{m}^{*}$, the Re[*K*(ω)] sign is negative, and the particles travel towards the low electric field gradient region, experiencing n-DEP effect [22].

## 3. Methodology

### 3.1. Device Design and Fabrication

**Figure 1.**
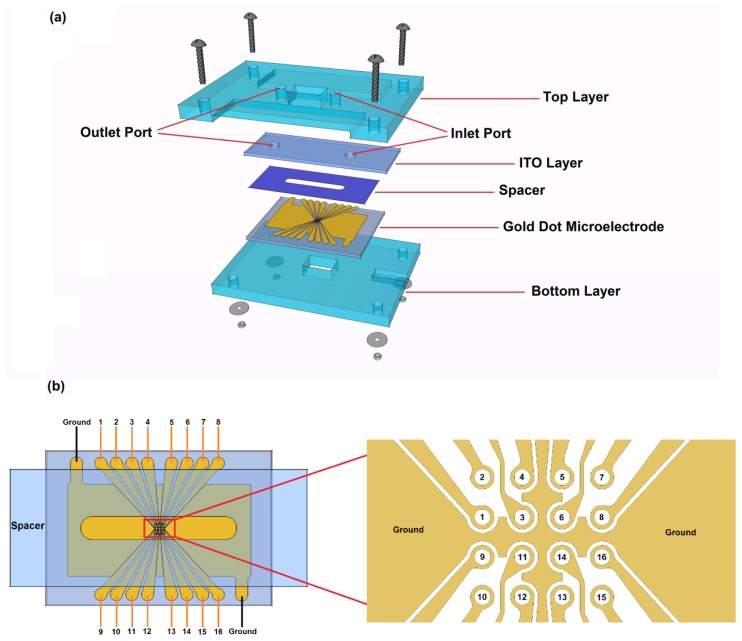
The LOC device design. (**a**) A schematic illustration of the proposed Lab-on-a-Chip device; (**b**) A high-resolution view of the proposed 4 × 4 microarray dot electrode. Sixteen individual inputs can be supplied simultaneously. Figure 1a reproduced with permission from [22].

The DEP device used in this work was first introduced in our recent review [22]. The device is composed of five layers, as illustrated in Figure 1a. The 4 × 4 microarray dot electrode (Figure 1b) was fabricated using standard photolithographic processes. Gold-coated glass slides (24K gold-coated microscopic glass slides, Fisher Scientific, Shah Alam, Malaysia) were cut into two halves, generating dimensions of 38 × 26 mm^2^. The microelectrode geometry was designed using AutoCAD software (Autodesk Inc, San Rafael, CA, USA), and the photomask was produced by JD photo-tools (Oldham, Lancashire, UK). The detailed fabrication processes have been previously described in [22].

Indium tin oxide (ITO)-coated glass slides (15–30 Ω ITO-coated glass slide, SPI Supplies, West Chester, PA, USA) serve as the ground electrodes and were cut to 45 × 15 mm^2^. Two 3-mm-diameter holes were drilled to provide inlet and outlet ports for microfluidic injections and ejections.

The top and bottom device covers were made of 4 mm thick polymethyl methacrylate (PMMA) plastic and were cut to 60 × 40 mm^2^ using a Computer Numerical Control (CNC) machine (VISION 2525, Vision Engraving and Routing Systems, Phoenix, AZ, USA). Rectangular holes (10 × 6 mm^2^) were introduced through the PMMA covers to allow monitoring the DEP events under the microscope.

The spacer, where the DEP effect occurs, serves as the gasket chamber. The spacer is a 0.1-mm-thick polyethylene terephthalate (PET) polyester sheet that was cut to a size of 38 × 15 mm^2^ using a cutter plotter (PUMA II, GCC, Xizhi District, New Taipei City, Taiwan). A 3 mm channel was introduced at the middle of the spacer to create space for the fluid to flow.

Flexible wires were soldered to the gold and ITO electrodes via silver-loaded epoxy and were connected to the function generator. Specific areas in the top and bottom PMMA covers were engraved to provide a space for these connections.

### 3.2. Sample Preparation

Particles with diameters of 1, 5 and 15 μm (microparticles based on polystyrene, 10%, Sigma-Aldrich, Munich, Germany) were used as the microparticles for manipulation by the proposed device. The aqueous microparticle suspensions of 1, 5 and 15 μm polystyrene particles were diluted with deionized (DI) water in volume ratios of 1:60, 1:40 and 1:20, respectively. The three suspensions were then ultrasonicated for 15 min to generate homogenous microparticle distributions. In each experiment, ten microliter of the particles suspension was pipetted into the spacer channel through the input port.

### 3.3. Experimental Setup

A function generator (GFG-8255A, Good Will Instrument, New Taipei City, Taiwan) was used to supply the microelectrodes with Alternating Current (AC) signal. The dynamic behaviors of the polystyrene microparticles in response to the DEP force were observed using an inverted microscope (BX51, Olympus, Shinjuku, Tokyo, Japan) with a CCD camera (UC30, Olympus) mounted on the top of the microscope. The images were captured and stored on a personal computer (PC).

## 4. Results and Discussion

During each DEP experiment, particle movements were observed in the microelectrode dot aperture area. Signals with different frequencies can be applied to individual dots simultaneously in order to achieve multiple microparticles separations concurrently; however, each figure presented in our results focuses on a single dot aperture to closely study the response of the particles to the DEP field inside the dot aperture.

**Figure 2.**
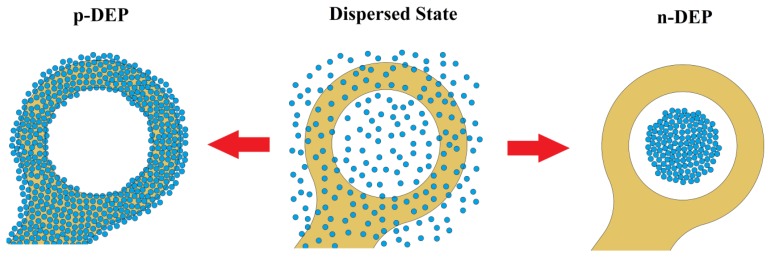
Schematic illustration for positive and negative DEP responses in the dot electrode.

In the case of a p-DEP response, particles travelled towards the high electric field gradient region (the dot edge) and away from the dot center, as shown in Figure 2. In contrast, in an n-DEP response, particles travelled away from the high electric field gradient region and collected at the dot center. This particle response to the DEP effect is aligned with DEP theory, microarray dot electrode simulations and previous DEP systems, in which particles are attracted to the electrode edge in p-DEP and repelled away from the electrode in n-DEP [26,38,39,45–47].

Furthermore, while observing particle migration in n-DEP, some of the microparticles were repelled from the electrode dots and collected between the dots. However, we focused only on dot centers because this is the confined area where cell characterization can be conducted by studying the change in light intensity inside the dot aperture.

First, we conducted our experiments using a 10 Vp-p sinusoidal signal; however, we observed that this signal amplitude was not sufficient to generate a rapid DEP response. We then increased the applied voltage to 20 Vp-p and observed a faster microparticle response (final particles position was reached in less than 5 s). Therefore, we used a 20 Vp-p sinusoidal signal for the rest of our experiments.

### 4.1. DEP Responses of 1, 5 and 15 μm Particles

We experimentally measured the crossover frequencies (the transition from p-DEP to n-DEP) of polystyrene microparticles in deionized (DI) water. The measurements were performed by increasing the frequency of the applied signal in small steps from a low value, at which all the microparticles of the same size clearly exhibited p-DEP, until the microparticles demonstrated an n-DEP response and were repelled to the dot centers. The crossover frequencies were 1100 ± 70 kHz and 180 ± 25 kHz for the 1 and 5 μm microparticles, respectively. However, the 15 μm particles exhibited an n-DEP in the frequency range of 1 kHz to 5 MHz. The obtained crossover values are in good agreement with the results reported by Khoshmanesh *et al.* [39].

**Figure 3.**
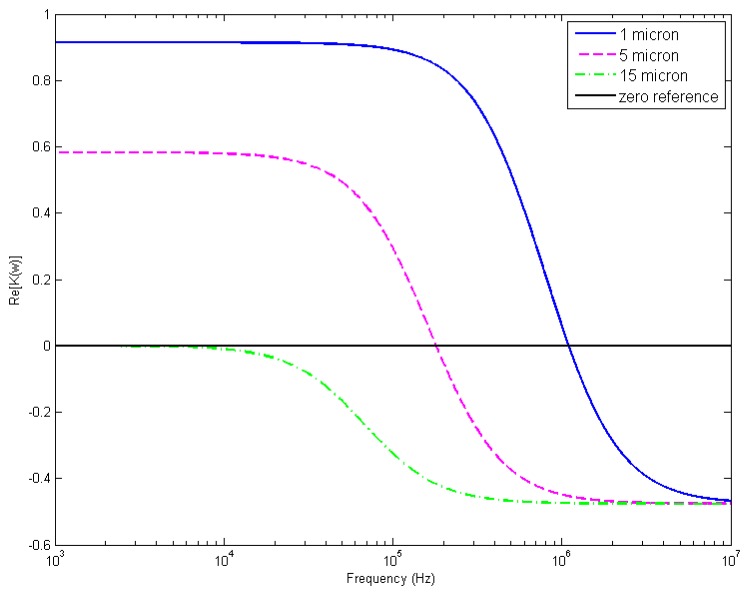
Re[*K*(ω)] *versus* frequency for 1, 5 and 15 μm polystyrene particles.

The DI water had a conductivity and relative permittivity of 2 × 10^−4^ S/m and 78, respectively. The relative permittivity of the polystyrene particles was 2.5 [39]. Using these data and the measured crossover frequencies, we calculated the overall conductivity of the particles using Equation (2) (provided that Re[*K*(ω)] = 0, since the crossover frequency occurs when the Re[*K*(ω)] changes its sign). The calculated overall conductivities of 1, 5 and 15 μm polystyrene particles equal to 4.6 × 10^−3^ S/m, 7.8 × 10^−4^ S/m and 2 × 10^−4^ S/m, respectively. Then, we applied these data in Equation (2) for a frequency range of 1 kHz to 10 MHz to obtain the Re[*K*(ω)] spectra (Figure 3).

### 4.2. Manipulation of 1 μm Particles

**Figure 4.**
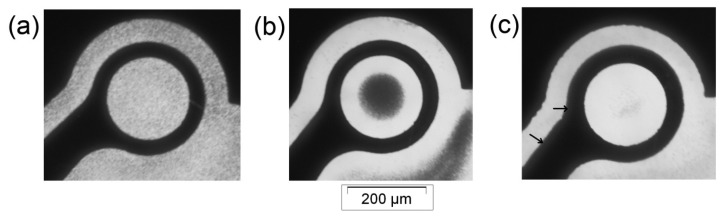
The manipulation of 1 μm particles under DEP field. The microparticles demonstrated p-DEP and n-DEP depending on the applied frequency. Microparticles (**a**) before applying a non-uniform electric field (homogenously distributed over the dot aperture); (**b**) after applying 5 MHz signal (n-DEP) and (**c**) after applying 100 kHz signal (p-DEP).

Figure 4 illustrates the 1 μm particle response when subjected to a DEP field. Before activating the device, the microparticles were homogenously distributed over the dot aperture, as shown in Figure 4a. A 5 MHz signal was then supplied to one dot; the result is depicted in Figure 4b. The microparticles were repelled towards the dot center. This microparticle behavior was in response to the n-DEP effect, in which the microparticles travelled away from the high electric field gradient region.

Furthermore, a 100 kHz signal was supplied to a different dot; the result is depicted in Figure 4c. In this case, the microparticles were cleared from the dot aperture and were attracted to the dot edge marked by the arrows (the attracted microparticles were unseen in the electrode black zone). This microparticle behavior was in response to the p-DEP effect, in which microparticles migrated towards the high electric field gradient region.

Clearly, the microparticle populations form a cloud at the dot center in the case of n-DEP. This cloud of microparticles can be used to quantify the DEP force applied on the microparticles by assessing the light intensity change in the dot aperture before and after applying the electric potential. In p-DEP, we do not expect to observe a change in the light intensity passing through the dot aperture after applying the electric signal, as no microparticles are obstructing light at the dot center.

### 4.3. Manipulation of 5 and 15 μm Particles

Moreover, the developed device was used to manipulate 5 and 15 μm particles. Equal volumes of 5 and 15 μm particle solutions, prepared as described in Section 3.2, were mixed and ultrasonicated for 15 minutes, to make a homogenous mixture of the microparticles.

**Figure 5.**
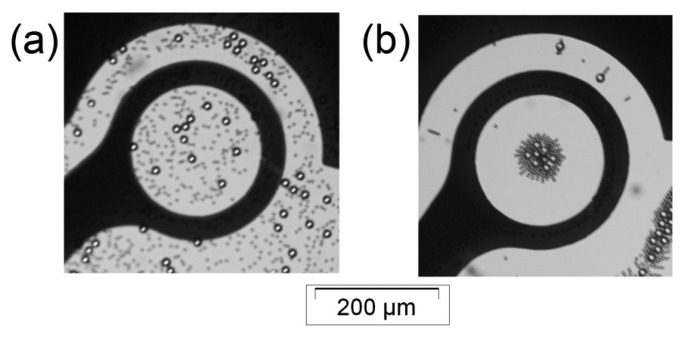
The manipulation of 5 and 15 μm particles under DEP field. The figure shows 5 μm particles (seen as the black points) and 15 μm particles (seen as the white circles) (**a**) before applying a non-uniform electric field (both sizes of microparticles were homogenously mixed and uniformly distributed) and (**b**) after applying 500 kHz signal (n-DEP on both microparticles).

Figure 5a demonstrates that both microparticle populations were homogenously mixed and uniformly distributed before applying the signal. Then, 500 kHz was applied to induce an n-DEP response from both microparticle sizes according to their DEP responses as plotted in Figure 2. Figure 5b exhibits the 5 and 15 μm particle n-DEP responses. Both microparticle populations were repelled from the electrode edges and collected at the dot center.

Furthermore, we observed that the 15 μm particles experienced a stronger DEP force than the 5 μm particles. This result was visible because the 5 μm particles surrounded the 15 μm particles which occupied the dot center. This phenomenon agrees with the particles' DEP responses shown in Figure 3; the 15 μm particles had a higher Re[*K*(ω)] value at 500 kHz compared with the 5 μm particles.

### 4.4. Separation of 1 and 5 μm Particles

**Figure 6.**
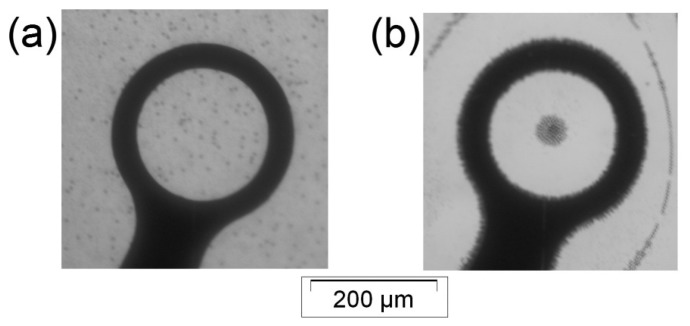
The separation of 1 and 5 μm particles under DEP field. (**a**) A uniform mixture distribution of 1 and 5 μm particles is depicted (1 μm particles were distributed over the entire electrode; the 5 μm particles were seen as the black points); (**b**) After applying 450 kHz, the 1 μm particles were attracted to the dot edge (p-DEP), whereas the 5 μm particles were repelled to the dot center (n-DEP).

In addition to particle manipulation, our proposed system was employed to separate microparticles with different sizes. First, 1 and 5 μm particles were separated using the DEP effect at a frequency of 450 kHz. A mixture of the two microparticle populations was prepared similar to that described in Section 4.3. Figure 6a depicts the mixture of 1 and 5 μm particles, in which the 1 μm particles were distributed over the entire electrode, and the 5 μm particles were seen as the black points. After applying the signal, we observed the 1 μm particle population to be repelled from the dot center and collected at the dot edge. In contrast, the 5 μm particles were repelled towards the dot center Figure 6b. These behaviors were expected and corresponded to the DEP responses of the microparticles (Figure 3). At 450 kHz, the 1 μm particles undergo p-DEP; conversely, the 5 μm particles exhibit n-DEP.

### 4.5. Separation of 5 and 15 μm Particles

**Figure 7.**
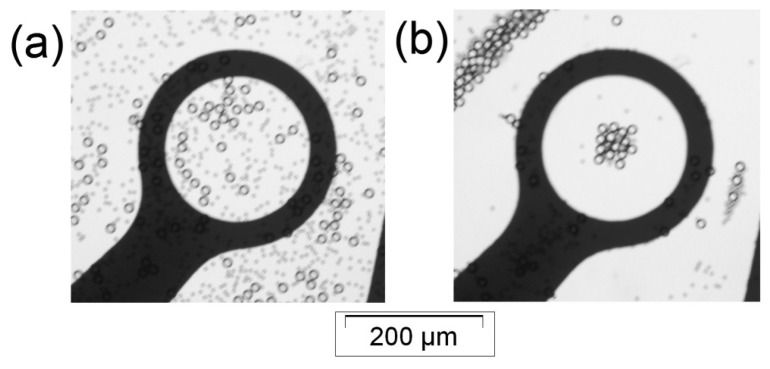
The separation of 5 and 15 μm particles under DEP field. (**a**) A uniform mixture distribution of 5 and 15 μm particles is shown. (**b**) After applying 12 kHz signal, the 5 μm particles were attracted to the dot edge (p-DEP), while the 15 μm particles were repelled to the dot center (n-DEP).

Our developed DEP system successfully demonstrated the separation of 5 and 15 μm particles. The samples were prepared similarly to those described in Sections 4.3 and 4.4. Figure 7a illustrates the mixture of 5 and 15 μm particles distributed over our dot electrode at the beginning of the experiment. The electrode dot was then energized with 12 kHz signal. The 5 μm particles were attracted to the electrode edge, leaving the dot aperture zone, while the 15 μm particles were repelled to the dot center (Figure 7b).

These microparticle migration behaviors are in agreement with their corresponding DEP responses, as shown in Figure 3. At 12 kHz, the 5 μm particles demonstrated p-DEP, while the 15 μm particles exhibited n-DEP.

We conclude from the previous results that our device successfully separated different size polystyrene particles (1 micron mixed with 5 micron, and 5 micron mixed with 15 micron) by using p-DEP and n-DEP effects. In principle, the larger size particle will be repelled and concentrated in the center of the dot electrode by n-DEP; however, the smaller sizes will be attracted and collected by the edge of the dot electrode by p-DEP. Hence, the different sizes could be successfully separated by a suitable frequency that needs simultaneously exhibited p-DEP or n-DEP for the different sizes. Even though, two different size particles can be individually separated by using the proposed dot electrode, such electrode geometry will not be able to separate three sizes individually in three separated regions. However, this device can be used to separate one size of particles from a mixture of different size particles provided that the target particles undergoes n-DEP and concentrated in the center of the dot electrode, while other populations simultaneously exhibit p-DEP and collected by the electrode edge.

The time needed to conduct microparticles manipulation and separation experiments were reduced significantly in the current project (5 s); compared to previous works, which used similar electrode geometries (4 min [48] and 1 min [29]). This is a result of the improvement in the electrode geometry and the LOC design.

Despite the fact that DEP experiments often require skillful personnel to perform, our proposed device can be used to measure experimentally the frequency-dependent dielectric properties of specific cells of interest, after separating them from a heterogeneous mixture of biological particles, without the need to take visual measurements by a skillful operator. This is feasible since the polarizability of the cells within the dot region can be directly related to change shifts in light transmission through the dot aperture, and quantified from analysis of digital images without the need for field mapping or image segmentation. Such on-chip parallel DEP experiments show great potential in the development of integrated DEP analysis systems where cells are manipulated, separated and characterized automatically without the need for highly technical human intervention.

## 5. Conclusions

A DEP system was developed, based on the work of Fatoyinbo *et al.* [37], to manipulate and separate microparticles using microarray dot electrodes. The dot electrode geometry features a well-defined and enclosed region of analysis, effective electric field penetration and an axisymmetrical electric field distribution. Our device showed a potential in manipulating and separating microparticles of different populations rapidly and efficiently. Microparticles migration was controlled merely by adjusting the applied frequency to induce p-DEP or n-DEP response on the target population in a mixture of various populations. In addition, the simple and rapid fabrication processes of planar electrodes make microarray dot electrode a potential solution to be utilized in research involving the development of cost-effective POC devices. In the future, such electrode geometries will be significant in developing DEP-based devices for rapid cells separation and characterization by implementing on-chip parallelization of DEP experiments.
